# P-1225. Aztreonam/Avibactam vs. Aztreonam Plus Ceftazidime/Avibactam for Multidrug-Resistant Stenotrophomonas maltophilia

**DOI:** 10.1093/ofid/ofaf695.1417

**Published:** 2026-01-11

**Authors:** Ashlan J Kunz Coyne, Rachel Gray, Alex Do, Elizabeth May, Ryan P Mynatt

**Affiliations:** University of Kentucky College of Pharmacy, Lexington, KY; University of Kentucky College of Pharmacy, Lexington, KY; University of Kentucky College of Pharmacy, Lexington, KY; University of Kentucky College of Pharmacy, Lexington, KY; University of Kentucky, Lexington, KY

## Abstract

**Background:**

*Stenotrophomonas maltophilia* is an opportunistic pathogen affecting vulnerable patients, including those with hematologic malignancies and chronic lung disease. Due to limited treatment options, outcomes remain poor. The IDSA recommends aztreonam plus ceftazidime/avibactam (ATM+CZA), while the recently FDA-approved aztreonam/avibactam (ATM/AVI) shows high in vitro activity. However, comparative efficacy data are lacking.

**Figure:**
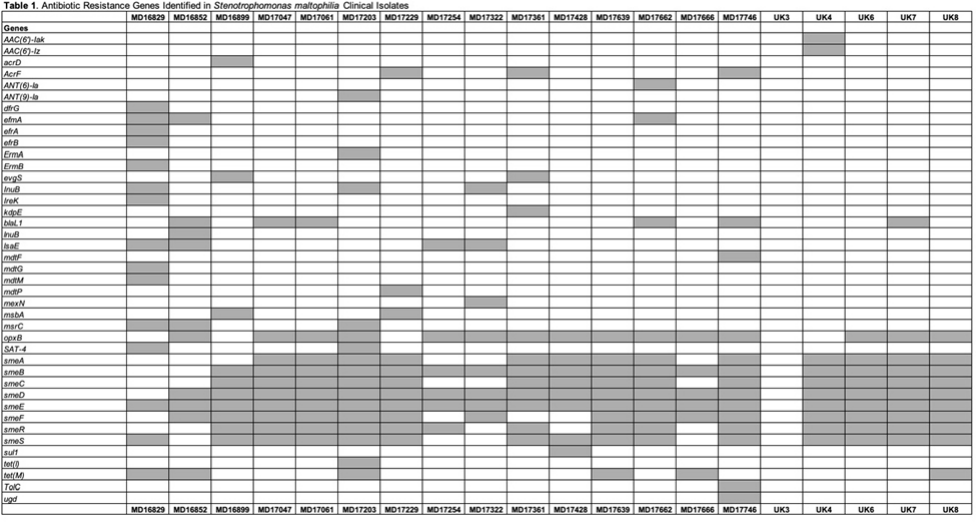

**Figure:**
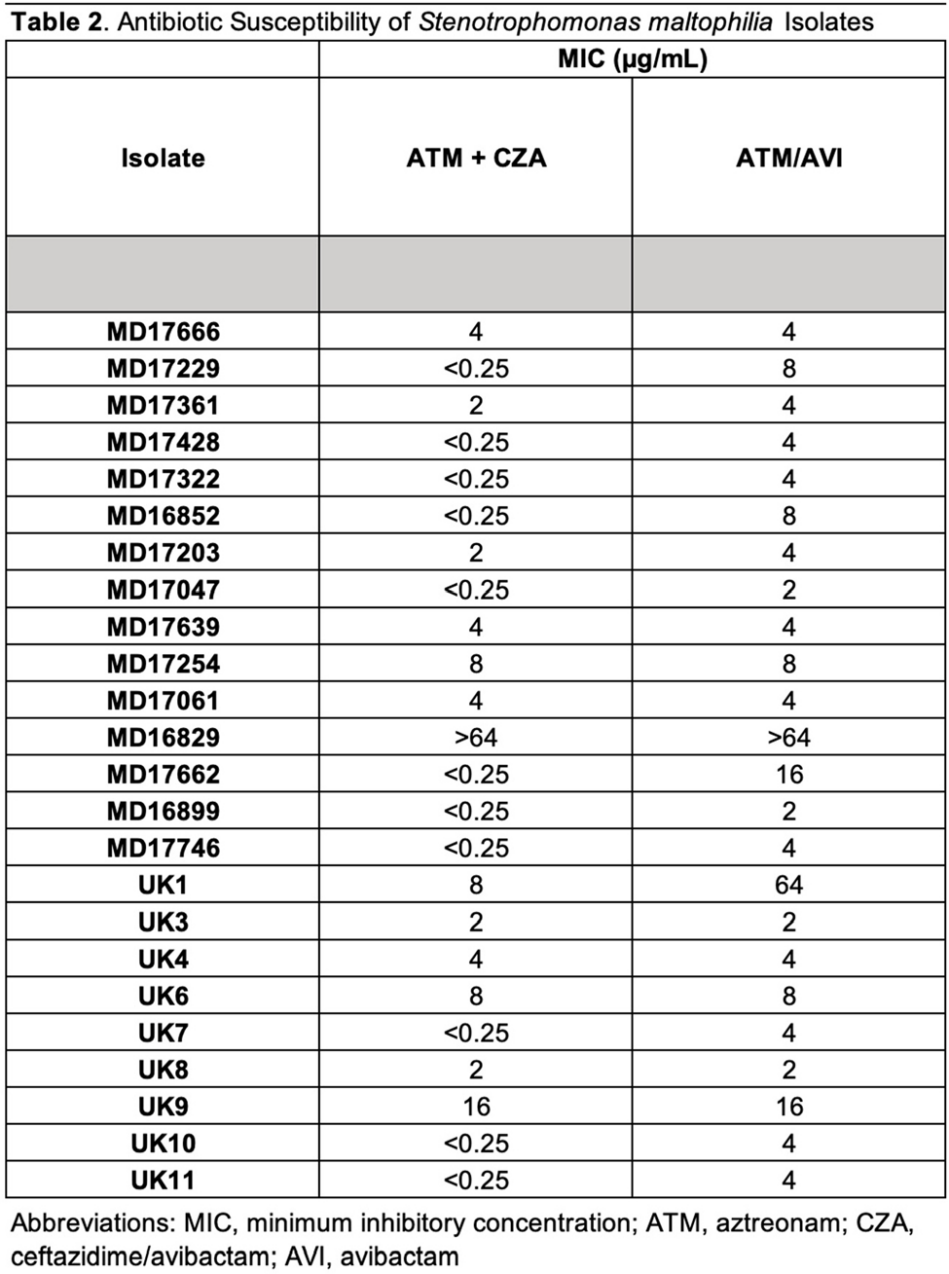

**Methods:**

We evaluated ATM+CZA and ATM/AVI against 24 clinical *S. maltophilia* isolates—15 from hematologic malignancy patients at MD Anderson (MD isolates) and 9 from chronic lung disease patients at UK Healthcare in Lexington (UK isolates). MICs were determined in triplicate by broth microdilution per CLSI guidelines; ATM/AVI MICs were verified using gradient diffusion strips (AbbVie). Time-kill assays were performed at clinically relevant Cmax concentrations of ATM, CAZ, and AVI. Bactericidal activity was defined as a ≥3-log₁₀ CFU/mL reduction from baseline. All isolates underwent whole-genome sequencing to characterize resistance mechanisms and provide genomic context for phenotypic findings (Table 1).

**Figure:**
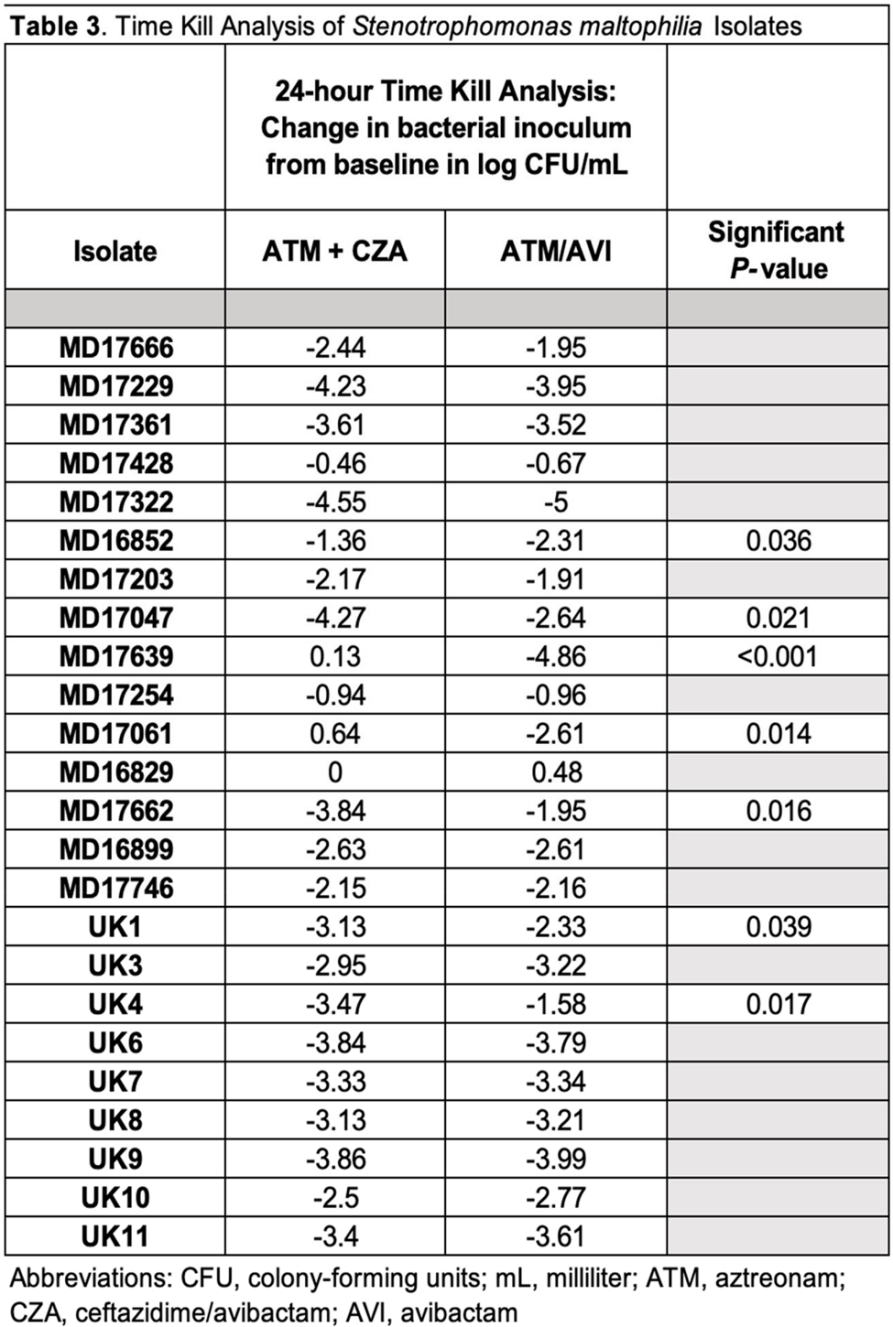

**Figure:**
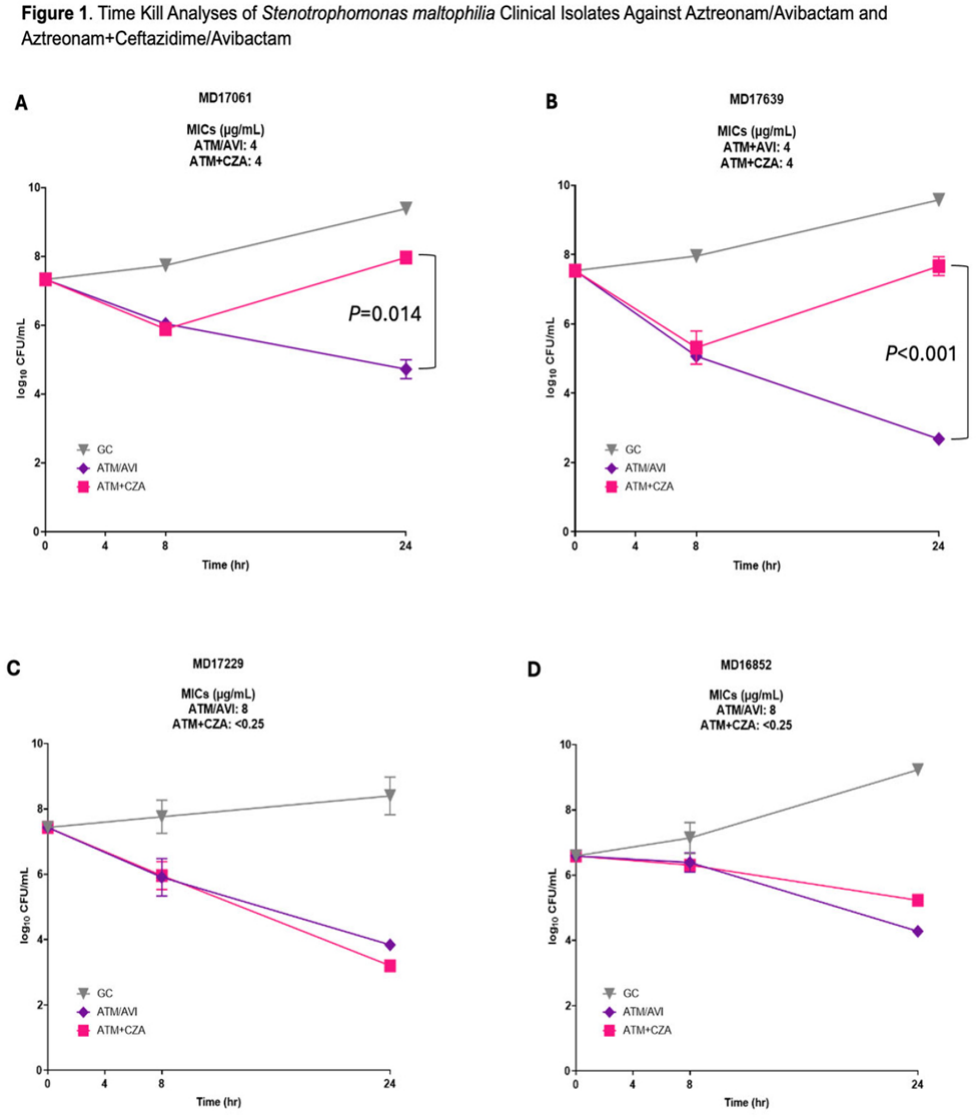

**Results:**

Among 24 *S. maltophilia* isolates, MIC₅₀/₉₀ values were 2/8 µg/mL for ATM+CZA and 4/16 µg/mL for ATM/AVI (Table 2). Time-kill analysis demonstrated bactericidal activity with both regimens across multiple isolates, though ATM/AVI showed more consistent killing overall (Table 3). Notable discordances between MIC and killing activity were observed. Isolates MD17061 and MD17639 had identical MICs (4 µg/mL) for both regimens, yet ATM/AVI produced significantly greater bacterial killing (P=0.014 and P< 0.001, respectively) (Figure 1A,B). Conversely, MD17229 and MD16852 exhibited large MIC differences (ATM+CZA< 0.25 µg/mL vs ATM/AVI 8 µg/mL) but similar bactericidal activity (Figure 1C,D).

**Conclusion:**

ATM/AVI demonstrated more consistent bactericidal activity than ATM+CZA, including in isolates with similar or less favorable MICs. Discordance between MIC and killing highlights the need for functional testing to guide treatment of multidrug-resistant *S. maltophilia*.

**Disclosures:**

All Authors: No reported disclosures

